# REASON CP: a systems-based framework for clinical reasoning to support participation across the lifespan in childhood-onset neurodisability

**DOI:** 10.3389/fpubh.2026.1800273

**Published:** 2026-04-21

**Authors:** Margaret Mayston, Sarah Foley, Kelly Reynolds, Willeke Walsh, Gillian Saloojee

**Affiliations:** 1 Neuroscience, Physiology & Pharmacology, Division of Biosciences, University College London, London, United Kingdom; 2 Kids Plus Foundation, Geelong, VIC, Australia; 3 Children's Therapy Services, Geelong, VIC, Australia; 4 Independent Practitioner, Melbourne, VIC, Australia; 5 Department of Physiotherapy, Faculty of Health Sciences, University of the Witwatersrand, Johannesburg, South Africa

**Keywords:** systems science, collaborative, holistic, ICF (International Classification of Functioning Disability and Health), transdisciplinary, family, participation, lifespan

## Abstract

Cerebral palsy, a common example of childhood-onset neurodisability (COND), is a heterogeneous neurodevelopmental condition characterized by complexity and variability across individuals. Children, families, and clinicians must navigate a maze of potential interventions, a challenge compounded by inconsistent quality of systematic reviews and lack of evidence for certain sub-groups, resulting in clinical decision-making uncertainty. Existing frameworks tend to focus on *what* intervention to use, rarely addressing the underlying reasoning processes that determine *why* a particular approach is most appropriate for a specific individual at a given point in time. This gap in clinical reasoning (CR) represents a significant barrier to optimizing outcomes for people with movement disorders.

REASON CP an acronym for Reflect, Evaluate, Activate, Support, Optimize, Navigate via Clinical reasoning for Participation was developed with the aim to address this gap. It is an innovative, structured, systems-based approach to CR, designed to support the selection of the right intervention for the right person at the right time, for the right reason. By integrating systems science with the International Classification of Functioning, Disability and Health (ICF), REASON CP is particularly well suited to the complexity of COND. By making the reasoning process explicit and collaborative, the framework strengthens shared decision-making between clinicians, families, and individuals, while supporting individualized, lifespan-oriented care across diverse settings.

By explicitly defining and operationalizing CR, REASON CP addresses a critical limitation in current practice, particularly the challenge of decision-making when robust evidence is unavailable. The framework supports individualized, family-centered care in any context and provides a transparent foundation for more relevant and rigorous research. A training module to support therapists in utilizing REASON CP in clinical practice is currently under development.

## Introduction

**Figure 1:**
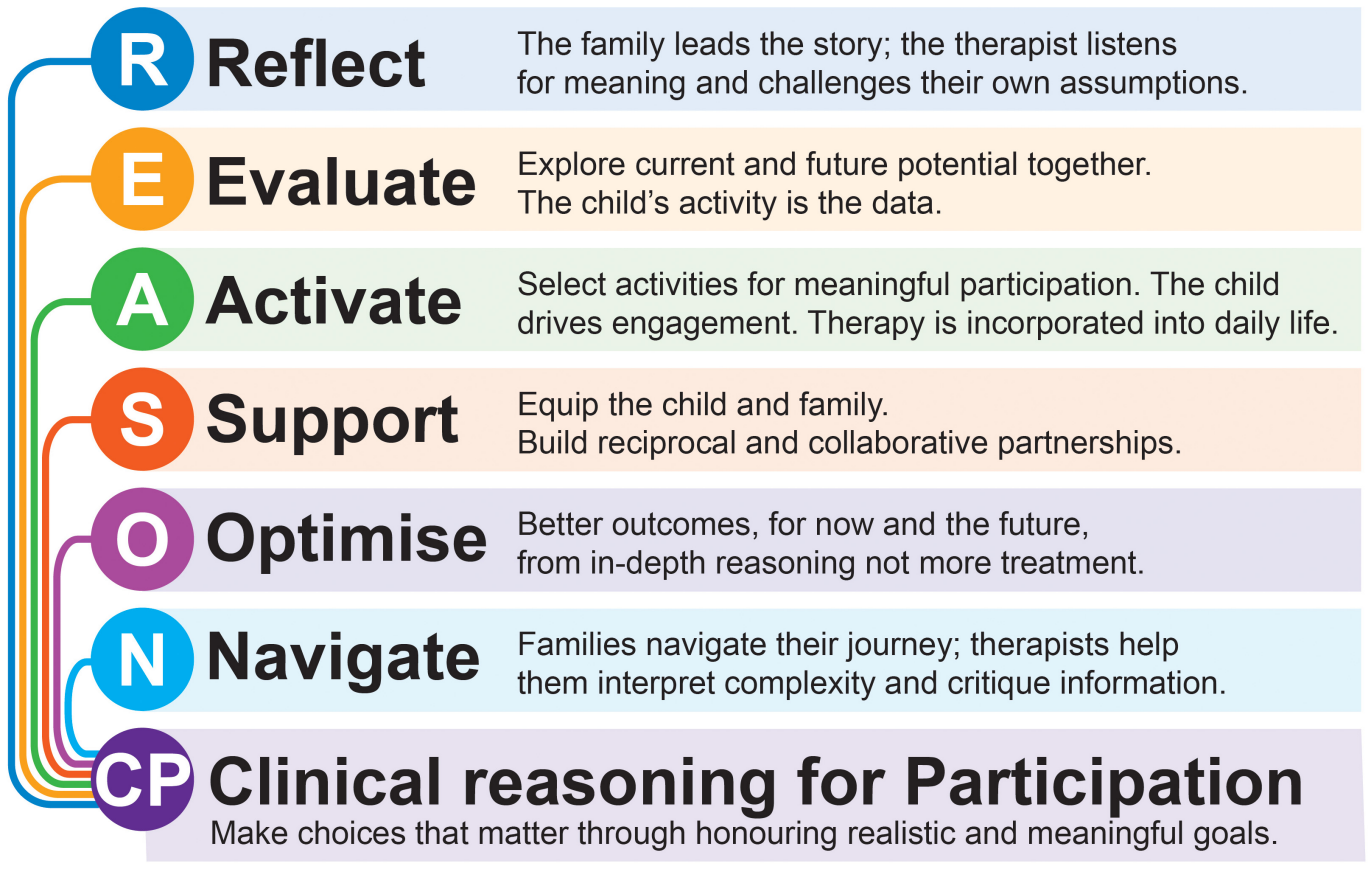
REASON CP applies its principles, listed on the left hand side, to collaborative working between child/family and therapists to ensure realistic goal setting now and for the future, comprehensive task analysis and interpretation, intervention implementation and evaluation based on applicable research evidence and underpinned by knowledge when it is absent. This achieves lifelong, meaningful participation.

REASON CP an acronym for Reflect, Evaluate, Activate, Support, Optimize, Navigate via Clinical reasoning for Participation, is an innovative, structured, systems-based approach to clinical reasoning that acknowledges the complexity of neurological conditions and the wide range of available interventions. As shown in Figure 1, it is a collaborative process between families/individuals and their therapy team to ensure the right intervention for the right child/ individual at the right time and for the right reason. REASON CP bridges the gap between goal setting and intervention selection, which is particularly important in situations where robust evidence is limited (1). It integrates relevant research evidence while also providing knowledge-based alternatives when evidence is unavailable, and can be applied across settings, resources, ages, functional levels, and when there are co-morbidities.

This paper describes REASON CP as a structured, collaborative, clinical reasoning framework for collaborative practice with families and individuals. From the outset, it emphasizes the development of a reciprocal relationship between therapist, child, and family as the foundation for effective collaboration. We demonstrate how REASON CP addresses the complexity of neurodisability, supports meaningful goal setting, and enables interventions to be tailored to each individual's context and stage of development. Central to the framework is understanding *why* an intervention is appropriate, rather than simply *what* intervention to use, while maintaining a forward-looking perspective. Although REASON CP is relevant for any individual with neurodisability, this paper focuses on childhood-onset neurosdisability (COND) and therefore refers primarily to the child and family.

## Goal setting to clinical decision-making

### Goal setting

REASON CP begins with building a relationship with the child and family through *Reflection*, where families share their story, hopes, and priorities. For example, in the section on information gathering these conversation prompts would include: **Therapist:** “*As part of our assessment of what X can do, I will be watching how X plays and moves; how X lets you know what X wants, and how you help X do everyday tasks like getting dressed and mealtimes”*. For GMFCS IV/V; families new to therapy: *What do you need help with? What would make caring for your child easier?* **Parent:** The favorite activity of X is indicated by the caregiver; *they spend most of their time e.g. playing on the floor*. Through active listening a shared understanding of what is meaningful is developed, fostering trust and two-way knowledge exchange that informs goal setting, clinical reasoning, and decision-making. A strong therapeutic relationship is recognized as an important contributor to positive outcomes (2).

Goal setting is widely regarded as the starting point of intervention planning for people with COND, yet its collaborative processes are poorly described in the literature. Families may have limited understanding of goal setting and variable confidence in clinicians' ability to support them (3–5), and it is often unrealistic to expect them to arrive with clearly articulated goals. Recent studies highlight the need for training and tools to support clinicians in constructing goals collaboratively with children and families (6, 7). In Uganda, families and therapists reported that collaborative goal setting was time consuming with initial goals often reflecting hopes for cure. Group discussions enabled these to evolve toward more realistic, participation-focused priorities over time. (8)

Although families recognize the value of goals for monitoring progress, many find the process emotionally demanding, particularly when considering future expectations for a child with COND. Families frequently rely on clinicians' expertise to help set realistic, meaningful goals, supported by reciprocal knowledge-sharing with clinicians combined with peer support from others with lived experience (9).

### Clinical reasoning

Clinical reasoning (CR) is neither well defined nor consistently described despite being considered integral to successful clinical practice (10–12). Most commentators agree that CR requires a strong knowledge base, critical thinking, reflection, and metacognition (thinking about one's own thinking), skills which take time to develop and are not easily acquired in undergraduate training (13). To address these challenges, REASON CP provides a structured, collaborative process to support effective CR and optimize participation in daily life (see Figure 3). Participation is defined as involvement across all spheres of life—home, school, work, recreation and community, through active engagement, belonging, and contribution (14).

Current models for cerebral palsy (CP) intervention do not provide clear guidance on the CR process (15–19). For example, Jackman et al. (16) propose observing task performance to identify limiting factors but do not explicitly describe how this should be undertaken. Furthermore, only five of the 13 guidelines promoted in this study were evidence based, with the remainder made on the recommendation of clinicians (16). The READ model (19) recommends further assessment when goals are unclear, without specifying the reasoning process. The absence of a structured and transparent approach places families and clinicians at risk of relying on incomplete or unreliable evidence (20).

REASON CP aims to address this gap by embedding CR within an evidence-based framework grounded in systems science and the International Classification of Functioning, Disability and Health [ICF; (21)], integrating four components of evidence-based practice: (i) research evidence, (ii) child and family preferences, (iii) clinician expertise, and (iv) contextual resources (22). This multidimensional perspective acknowledges individual abilities, aspirations, and environments, supporting more individualized interventions. Explicit discussion of *why* interventions are chosen is central to shared, context-sensitive decision-making with families and individuals.

### Research evidence

Evidence alone is often insufficient, particularly in pediatrics where development and environmental factors confound intervention effects. Much of the existing literature focuses on children with unilateral CP, and those at GMFCS levels I–III, leaving substantial gaps for those at higher levels, with bilateral presentations, co-morbidities, or living in remote and low-income settings with limited access to therapy, early intervention, surgery, or pharmacological care. Evidence is also limited for supporting transitions into adolescence, adulthood, and employment (23, 24). In addition, the variable quality of systematic reviews further limits their clinical utility (25).

Systematic reviews summarize best available evidence but should not be interpreted as prescriptive guides to practice (26), as they can “inform but not replace sound clinical reasoning” (27). Families and clinicians must therefore interpret evidence judiciously and integrate it with real-world considerations, a particular challenge for less experienced therapists. Therapists are often working in highly challenging, complex contexts, such as for a child with severe dystonia and no verbal communication, or children with significant co-morbidities or sensory impairments affecting task performance and learning, particularly in remote settings and low- and middle income countries (LMIC), where for instance a teenager with fixed contractures for whom correction is unavailable. REASON CP provides a clear framework to support this process, ensuring clinical reasoning is comprehensive, shared, participation-focused, and is evidence-based where evidence exists and knowledge-informed where it does not. Established bodies of knowledge including child development, family- and person-centered practice, neuroscience, and muscle physiology underpin intervention selection when robust evidence is lacking.

### Clinical decision-making

The preceding discussion highlights the need to place greater emphasis on effective goal setting and clinical reasoning within the clinical decision-making (CDM) process. REASON CP uses in-depth clinical reasoning to determine *why* an intervention is appropriate, and supports realistic and meaningful intervention decisions that address the complexity of COND, often addressing multiple goals within one activity.

## Theoretical basis of REASON CP

### Systems science and systems thinking

The theoretical foundation of REASON CP lies in the application of systems science and systems thinking (28), which recognize that outcomes in complex situations arise from interdependent, non-linear interactions among multiple variables. CP exemplifies this complexity: impairments in motor control, muscle tone, sensory processing, and cognition interact with contextual and environmental factors to influence participation. Linear approaches which dominate many research and intervention models fail to capture these realities (29) limiting their application to real-life.

Systems thinking offers tools such as systems maps to visualize interconnections across domains (20, 30). For example, surgical correction of musculoskeletal malalignment may improve biomechanics, but without addressing proprioceptive, sensory, and motor control systems it may inadvertently reduce walking performance and confidence (20; ch.4). By highlighting interdependencies, a systems approach supports more comprehensive planning, holistic interventions, and potentially improved outcomes (30). This shifts the focus from impairments to the more important interconnections which enables effective CDM (20).

**Figure 2:**
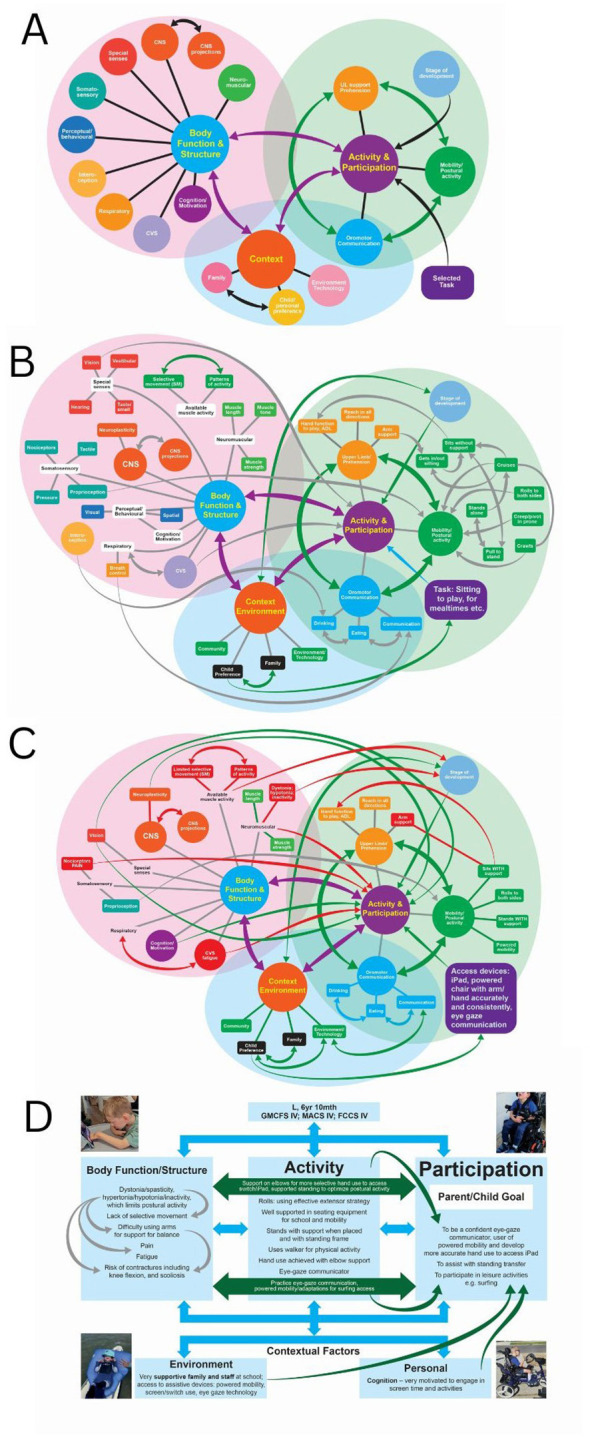
Map **(A)** shows the basic domains and sub-domains of the ICF which are used to build systems map **(B)**, which shows most of the possible interactions between the ICF elements, many of which are interdependent, particularly the 3 main streams of development: mobility/postural activity; arm/hand function and oromotor/communication. Map **(C)** represents the child in the case study where the main elements and their interconnections are outlined: green denotes the facilitators and red the barriers to achievement of the task goal. Panel **(D)** shows how the findings of the systems map are translated into the ICF format domains, all of which are interdependent. Abbreviations: ADL: activities of daily living; CNS: central nervous system; CVS: cardiovascular system; SM: selective movement; UL: upper limb

The systems maps are structured around the domains of the ICF, which provide the starting point for their construction (Figures 2A–D). Firstly, the interrelationships among the main ICF domains—context, activities and participation, and body functions and structures are mapped (Figure 2A). Interactions among specific components are then added to demonstrate the complexity of COND (Figure 2B). Key areas of concern are identified for each individual (Figure 2C; case study) and translated into an ICF summary (Figure 2D).

Together, these support collaborative discussion with families, enhance understanding of current and future possibilities and guide intervention selection within the individual's context. The systems map also offers a way to identify points likely to bring about modification of one or more systems, often simultaneously, resulting in more effective system outcome (31). These are further discussed in the case study.

### Integration of systems thinking with the ICF

When integrated with the International Classification of Functioning, Disability and Health (ICF), systems science highlights the interdependence of its domains. The ICF emphasizes contextual domains, environmental and personal factors, that interact with body functions and structures and activities to shape participation. REASON CP operationalises this model by directing clinicians to consider these interactions rather than impairments alone, identifying barriers and facilitators within the child's environment and supports. Participation is conceptualized as the “system outcome,”—an emergent property arising from interactions within the system—reflecting meaningful involvement in daily life across individual contexts.

This integration is illustrated in Figure 2D, where the emphasis is on the interdependence and interrelationships across all ICF domains.

## Developmental trajectories and the importance of challenge

Because COND is developmental, it must be considered within the broader context of all aspects of development. Therapists have a responsibility to promote progression based on what is possible, informed by neuroscience and developmental principles. However, maintaining a skill or level of function may sometimes be an appropriate goal, particularly during periods of rapid growth or transition (e.g. starting school or employment) or when regression may occur.

REASON CP emphasizes that interventions should both consolidate existing abilities and challenge children to extend their competencies. Development involves ongoing challenge that drives progression across neural, musculoskeletal, motor, social-emotional, cognitive, and behavioral domains.

Neuroplasticity, driven by activity-dependent processes in the developing central nervous system (32), persists throughout life but is greatest in early childhood. Bone growth and peak mass acquisition continue into the second decade (33), and central nervous system development extends through adolescence and into adulthood (32). Clinicians therefore have a responsibility to support skill development across the lifespan to promote current and future participation.

**Figure 3:**
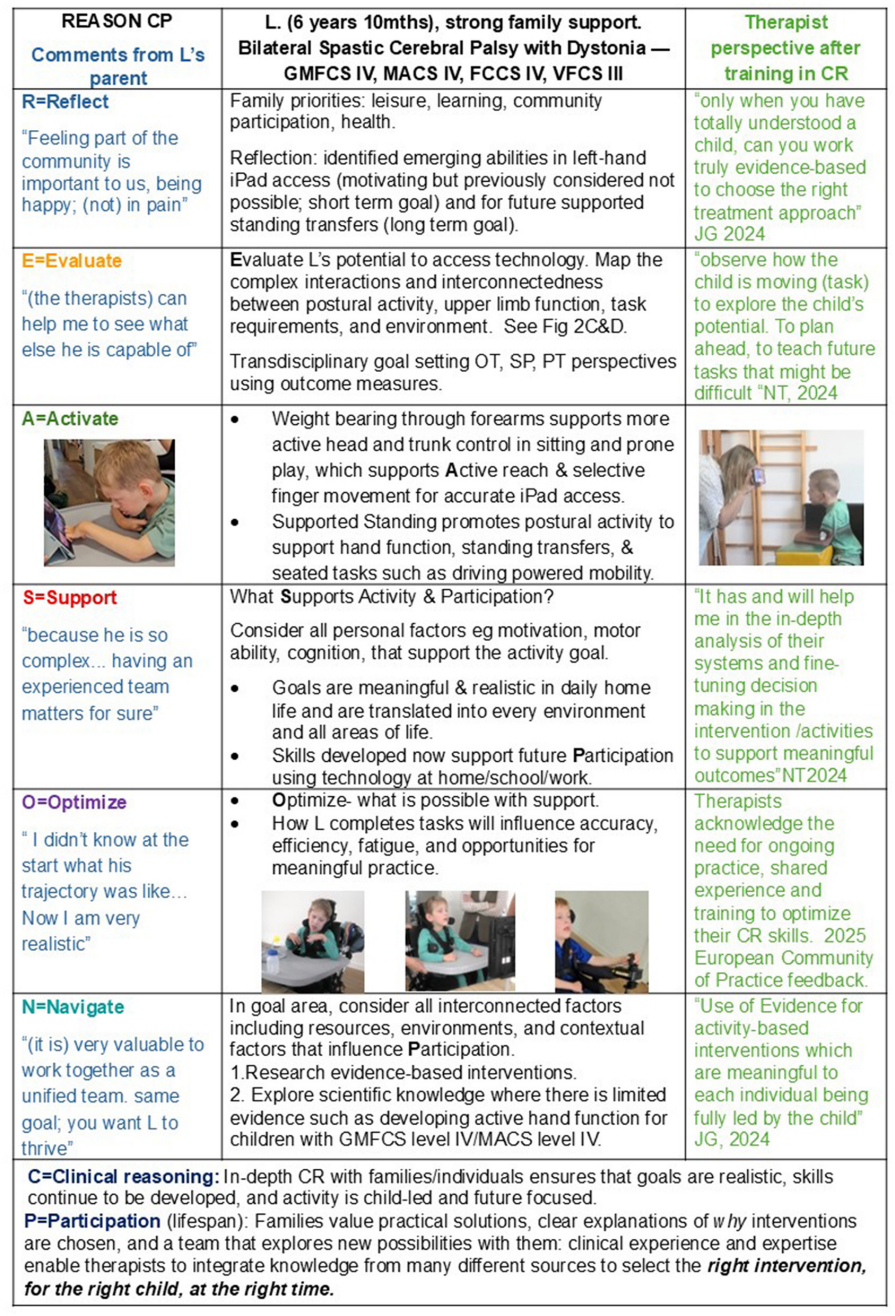
A case study which demonstrates how REASON CP is applied to child/family and therapists to enable collaborative working and a truly holistic approach. The first column shows each of the REASON CP components with a comment from L's parents. Column 2 shows the main observations and their interpretation, while column 3 provides feedback from therapists who have undertaken training in clinical reasoning.

REASON CP considers future potential by integrating current abilities with anticipated developmental trajectories and contextual factors. As families often seek guidance about their child's future, clinicians must support realistic expectations. In the case example (Figure 3), task analysis identified potential for functional hand use, enabling participation through technology now and supporting future access to assistive technologies. This lifespan perspective distinguishes REASON CP by supporting progression, adaptability, and long-term participation.

## Families and contextual resources

REASON CP can be applied across diverse contexts and environments, regardless of the availability of technology, equipment, or assistive devices. Families are the most influential environmental factor shaping outcomes for children with CP, and the reciprocal relationship between child and family must be considered (34). In many settings, particularly remote and/or resource-limited contexts, families are the primary opportunities for enrichment and developmental support. REASON CP recognizes this by incorporating contextual resources as a core component of clinical reasoning to enable adaptation to each circumstance.

For parents, tension may exist between parenting roles and supporting intervention implementation (35). Therapy teams must therefore be sensitive to the demands of daily life and prioritize time-efficient, effective interventions, which should be compatible with family routines, promote participation in everyday activities, and enhance enjoyment of life, rather than being perceived as exercises that “must be done.”

Through shared reasoning, clinicians and families develop a common understanding of current abilities, aspirations, and future trajectories, which Supports realistic goal setting, strengthens family-centered care, and ensures interventions are feasible and sustainable. It also Supports Navigation of the range of available interventions to identify contextually appropriate solutions that promote Optimal activity and Participation in daily life.

## Teamwork and transdisciplinary practice

Effective clinical reasoning depends on teamwork. Although multidisciplinary and interdisciplinary approaches are common, they may reinforce professional boundaries and limit cohesion in goal setting and intervention planning. In contrast, REASON CP promotes a transdisciplinary approach in which core knowledge and skills, about mobility/postural activity, upper limb function, and communication, are shared across disciplines. The case study illustrates how shared competencies support holistic care: for example, accurate eye-gaze use requires consideration of postural activity, communication needs, and access to technology across environments. This model reduces fragmentation and is highly valued by families (36) and is suggested to lead to better co-ordinated services (37).

The authors' experience demonstrates the utility of transdisciplinary practice in both high- and low-resource settings. In contexts where specialist expertise is limited, particularly in LMICs and remote areas, shared competencies enable a single clinician to address multiple domains of functioning, ensuring integrated and consistent support despite constrained resources. Transdisciplinary teamwork therefore promotes both efficiency and equity in care delivery.

## Application of REASON CP in a real-world setting

### Case study

The case study illustrates application of REASON CP through detailed task analysis of the child and family's goal activities, demonstrating that barriers to accurate hand function for iPad access could be addressed. The 9-step clinical reasoning process developed from the REASON CP team's collective clinical, teaching, and lived experience, guides therapists from establishing collaborative partnerships to identifying barriers and facilitators for effective clinical decision-making.

Analysis of interacting systems—particularly neuromuscular factors (dystonia, inactivity) and somatosensory factors (pain)—together with developmental knowledge, identified two key leverage points (31) capable of influencing system outcomes, in this case participation. Practicing standing transfers promoted trunk activity and supported upper limb/hand function. In addition, applying the developmental principle of elbow support to sitting, enabled L to practice more accurate and consistent hand use to access devices.

Practicing meaningful daily activities with appropriate challenge supports development within current capabilities and future participation, including independent digital environmental control (see Figures 2C–D and Figure 3).

## What's next?

### Knowledge translation: training

Clinical research reports average outcomes, which may not always translate directly to individuals (20). As noted by Gough & Shortland (20), clinicians must sometimes question guidelines and use their understanding of the person to consider alternative approaches. In complex conditions, non-linear thinking relies on experience, intuition, and knowledge of interacting systems, supported by expert clinical reasoning and communication among professionals, with the child and family central to and involved in decision-making (30, 38). There is increasing recognition of the need for training in childhood-onset neurodisability (COND) that promotes meaningful participation, functional development, and a lifespan perspective (37). These competencies form the basis of the REASON CP training module currently under development.

Knowledge translation is rarely a simple transfer of research into practice (39). Applying evidence effectively requires experience, confidence, and critical thinking—key elements of clinical reasoning (39). Managing complex conditions such as cerebral palsy therefore requires sustained professional development. Less experienced therapists benefit from structured guidance, while experienced clinicians must continue to critically reflect rather than rely solely on intuition (40, 41).

REASON CP provides a structured clinical reasoning framework that emphasizes collaborative working and case-based learning. It supports therapists to articulate decision-making processes with families and apply existing and emerging evidence to complex or unfamiliar presentations. Rather than focusing on discrete assessments or treatment protocols, REASON CP emphasizes integration of knowledge and shared reasoning. Therapists are guided to reflect and evaluate with families what a child can do, can nearly do, can do with assistance, and what they wish to achieve. The nine-step framework prompts clinicians to listen to families and operationalise collaborative clinical reasoning.

The REASON CP training module is being co-developed as an online transdisciplinary programme with people with lived experience to ensure accessibility and relevance. It aims to equip therapists to build collaborative relationships with families, determine current and future potential, critically apply research evidence, plan interventions when evidence is limited, and work holistically across settings. Preliminary feedback suggests increased confidence in family engagement and clinical decision-making, with participants describing the framework as systematic and logical, and is informing the development of the training module. These findings highlight the need for structured clinical reasoning training to support transparent, well-reasoned, and effective care.

## Evaluation of REASON CP

REASON CP is a new framework and therefore requires robust evaluation to determine its usefulness for families and therapists. Evaluation is already underway and is informing the development of the training module. Once the training module is complete, it will be formally evaluated to assess its effectiveness in supporting clinical reasoning across different therapy disciplines and practice settings. Although the current focus is on CP, REASON CP can be readily applied to other movement disorders, providing therapists with broad caseloads or those working with adults a structured tool to support clinical reasoning and effective clinical decision-making across contexts.

### Implications for research

REASON CP also offers guidance for research. Traditional reductionist study designs, which focus on linear relationships, are increasingly recognized as inadequate for capturing the multidimensional nature of CP interventions (29). Systems-informed, non-linear research designs may better reflect real-world practice, supporting evidence that is both rigorous and clinically relevant (42). By explicitly defining clinical reasoning, REASON CP provides a framework to bridge clinical practice and research, ensuring future studies align with the multifactorial realities of neurodisability. Gough & Shortland (20) support this idea by stating that the focus on “optimizing the child's experience of their world through their lived-in body may allow this clinical evidence to be more easily apprehended and considered by the child and family”—and the therapists.

## Conclusion

REASON CP represents an innovative and practical framework for clinical reasoning in developmental and other movement disorders. By focusing on *why* interventions are chosen rather than simply *what* is available, it provides a structured, transparent, and collaborative approach to decision-making. Grounded in systems science and integrated with the ICF, it emphasizes participation as the emergent outcome of effective, individualized care. The framework supports families and individuals, promotes developmental progression and meaningful goals, and advocates transdisciplinary practice, making it applicable across diverse contexts.

By explicitly defining and operationalising clinical reasoning, REASON CP addresses a significant gap in current practice. It fosters individualized, family-centered care while also providing a foundation for more relevant and rigorous research. The goal of REASON CP is to offer a pathway to optimizing interventions for all children and young people with CP, ensuring the right support is provided at the right time and for the right reasons right across the lifespan.
